# Supplementary material for: Alignment-free comparison of metagenomics sequences via approximate string matching
Source: Bioinform Adv. 2022 Oct 21;2(1):vbac077. doi: 10.1093/bioadv/vbac077 (PMC9645238; doi:10.1093/bioadv/vbac077)
Supplement: vbac077_Supplementary_Data [file vbac077_supplementary_data.pdf]

Supplementary Data

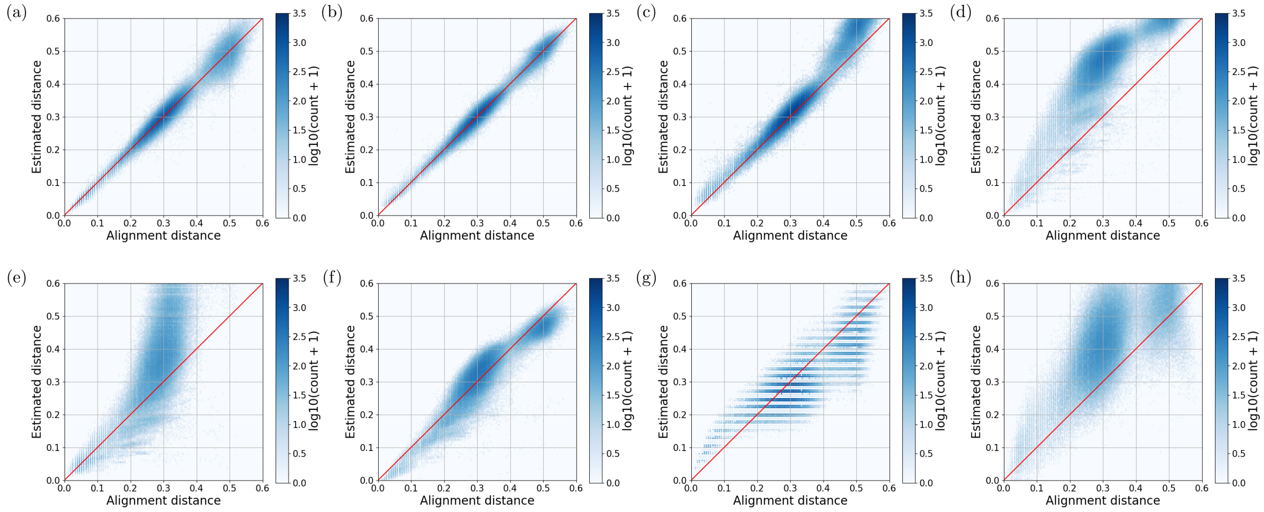

Fig. 1. Visualization of alignment distances versus estimated distances computed by eight methods: (a) AsMac, (b) SENSE, (c) NeuroSEED, (d) ACS, (e) Kr, (f) kmacs (k=2), (g) k-mer (k=3), and (h) FFP (l=9), performed on the Qiita dataset. Each dot represents a sequence pair, and the color of a hex bin represents the number of sequence pairs in the bin. The number in a parenthesis is the parameter that achieved the best result for the corresponding method.

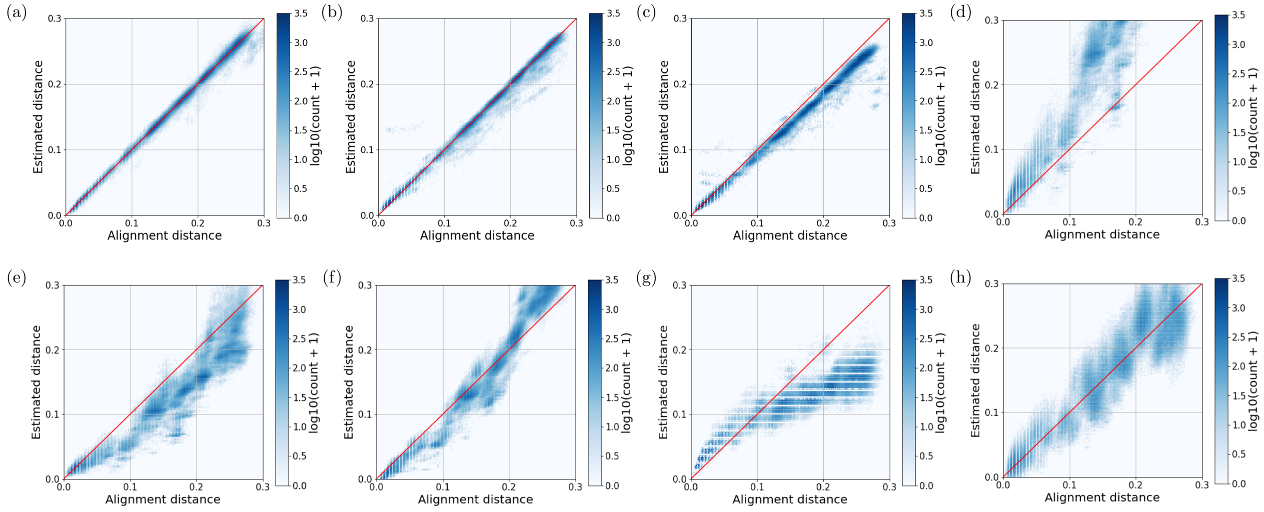

Fig. 2. Visualization of alignment distances versus estimated distances computed by eight methods: (a) AsMac, (b) SENSE, (c) NeuroSEED, (d) ACS, (e) Kr, (f) kmacs (k=2), (g) k-mer (k=3), and (h) FFP (l=10), performed on the RT988 dataset.

© The Author 2022. Published by Oxford University Press. All rights reserved. For permissions, please e-mail: journals.permissions@oup.com

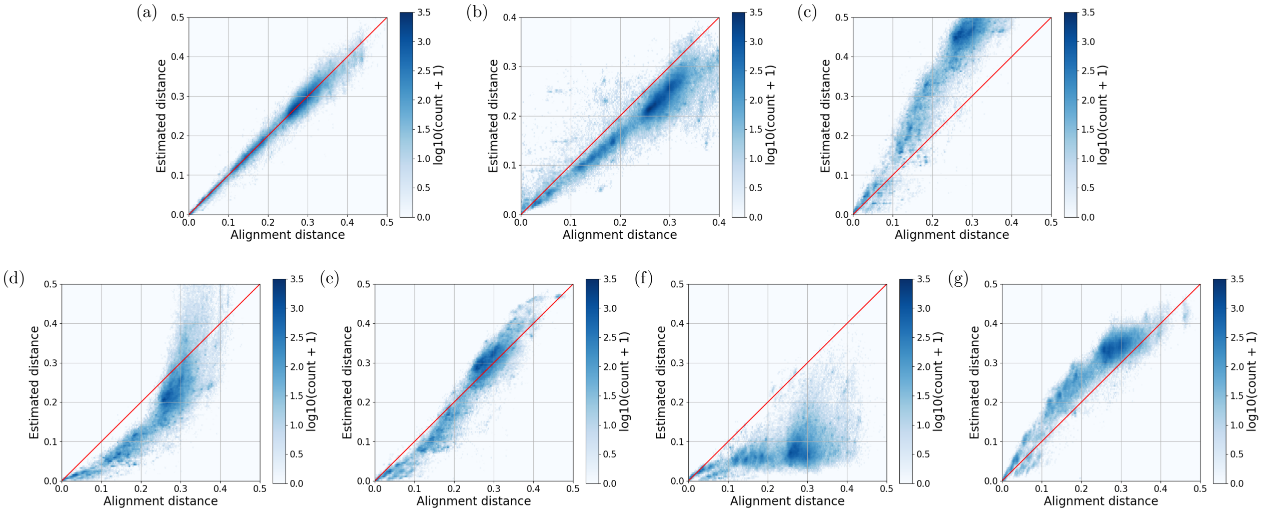

Fig. 3. Visualization of alignment distances versus estimated distances computed by seven methods: (a) AsMac, (b) NeuroSEED, (c) ACS, (d) Kr, (e) kmacs (k=3), (f) k-mer (k=6), and (g) FFP (l=13), performed on the Silva-23S dataset.

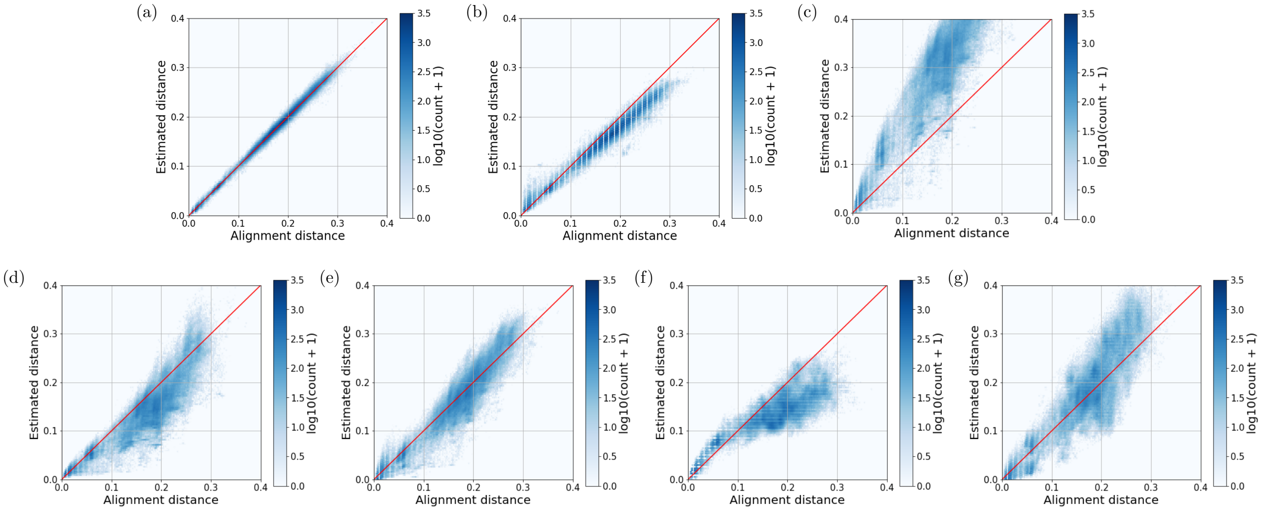

Fig. 4. Visualization of alignment distances versus estimated distances computed by seven methods: (a) AsMac, (b) NeuroSEED, (c) ACS, (d) Kr, (e) kmacs (k=2), (f) k-mer (k=5), and (g) FFP (l=12), performed on the Labonte lake dataset.
